# Integrating antibody and infection-based testing into trachoma surveys

**Published:** 2024-10-02

**Authors:** Sheila West, Titus Watitu, Laura Senyonjo, Jeremiah Ngondi, Timothy Jesudason

**Affiliations:** 1 El-Maghraby Professor of Preventive Ophthalmology and Vice Chair for Research, Wilmer Eye Institute, Johns Hopkins University.; 2 Preventive Chemotherapy Manager, Ministry of Health, Nairobi, Kenya.; 3 Sightsavers, Haywards Heath, United Kingdom.; 4 Senior Epidemiologist: RTI International, Cambridge, United Kingdom.; 5 Special Projects and Campaign Partnerships: International Coalition for Trachoma Control, London, United Kingdom.

**Testing for antibodies enables programmes to detect previous exposure to *C. trachomatis*.**

**Figure:**
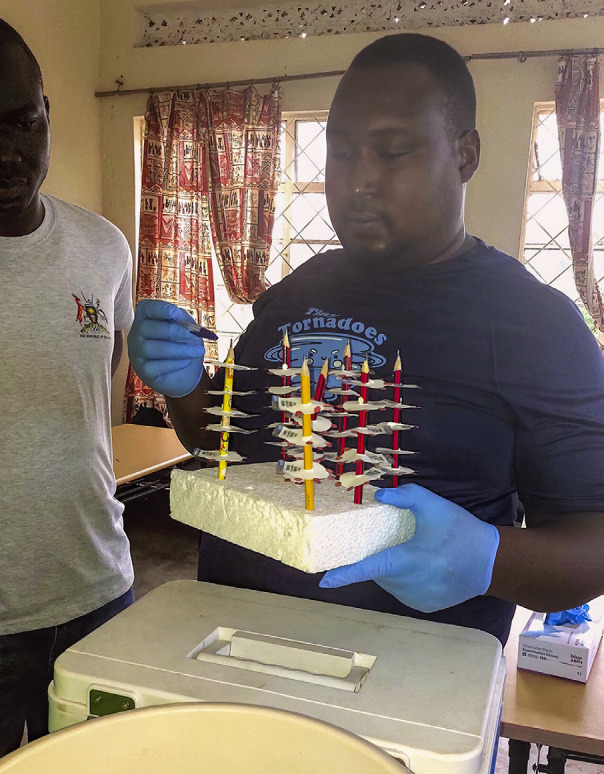
Dried blood samples are archived for later analysis during trachoma survey field work. UGANDA

© JEREMIAH NGONDI, RTI INTERNATIONAL, SIGHTSAVERS CC BY-NC-SA 4.0

Trachoma, the world's leading infectious cause of blindness, is caused by repeated episodes of infection with *C. trachomatis*. Trachoma is targeted for global elimination as a public health problem by 2030. The World Health Organization (WHO) defines elimination as a public health problem, in part, by a prevalence of trachomatous inflammation—follicular (TF) in children aged 1–9 years of <5% in each endemic district.

To estimate the prevalence of TF in children, programmes typically deploy field workers, which are trained to grade TF using a 2.5x magnifying loupe and a flashlight. This approach is becoming more difficult as the global prevalence of TF declines and fewer people are affected, which reduces the opportunities available for training and intergrader agreement assessment.

Furthermore, despite many rounds of MDA, an increasing number of districts have been identified as having persistent or recrudescent active trachoma. This has led many national trachoma programmes to evaluate the potential of additional indicators to supplement clinical trachoma grading, including testing for antibodies to *C. trachomatis* (serology) and testing for ocular infection. Testing for antibodies enables programmes to test for previous exposure to *C. trachomati*s and, when combined with age data, can be used to estimate the transmission of infection over time. Infection testing, on the other hand, provides direct evidence of current *C. trachomatis* infection.

In Kenya, the Ministry of Health has been conducting operational research since 2021 to better understand trachoma epidemiology, particularly in areas identified as having persistent and recrudescent active trachoma. Using these alternative indicators has enabled the national programme to identify areas with high reinfection following mass drug administration and geographical areas with high transmission, which have led to programmatic adaptations, including improvements to mass drug administration, such as increasing from annual to biannual distribution.

To test for infection, field workers are trained to collect ocular swabs from children aged 1–5 years old by swabbing the upper tarsal conjunctiva of the eye. Ocular swabs are placed into a tube, kept cool in the field and stored frozen until the time they are tested by trained laboratory personnel using commercially available and sensitive PCR methods. To test for antibodies, field workers prick a child's finger and collect 60 μl of blood onto a filter wheel. The spots of blood are air-dried and frozen until tested for antibodies by trained laboratory personnel.

Recognising that trachoma is targeted for elimination as a public health problem by 2030, Kenya's Ministry of Health is also strengthening the capacity of its eye health system to accurately identify and manage recrudescence of trachoma in a post-elimination setting. This includes establishing regional laboratories to conduct serology and infection testing as well as repurposing laboratories that were established to support the country's COVID-19 response. Strengthening the country's laboratory system has benefits beyond trachoma, with the laboratories being able to identify other endemic diseases.

Experiences from Kenya are consistent with several other countries, including Cameroon, Ethiopia, Mozambique, Nigeria, Tanzania, Uganda, and Zambia, which are conducting operational research on serology and infection for estimating ongoing transmission. Notably, complementary indicators can provide more reliable information about the number of azithromycin donations needed and support decisions about modifying MDA programmes to target persistent and recrudescent areas of active trachoma, as seen in Kenya, Mozambique, and Tanzania. These indicators have also provided evidence to validate the elimination of trachoma as a public health problem, as seen in Ghana and Vanuatu.

As the evidence base for using these serology and/or tests of infection increases, it will be important to have standardised processing and data analysis methods to inform survey planning and to guide the appropriate use of these indicators in determining programme activities.

